# Hypoxia-Inducible Factors as an Alternative Source of Treatment Strategy for Cancer

**DOI:** 10.1155/2019/8547846

**Published:** 2019-08-14

**Authors:** Musbau Adewumi Akanji, Damilare Rotimi, Oluyomi Stephen Adeyemi

**Affiliations:** ^1^Department of Biochemistry, University of Ilorin, Ilorin, Nigeria; ^2^Department of Biochemistry, Medicinal Biochemistry, Nanomedicine & Toxicology Laboratory, Landmark University, Omu-Aran 251101, Nigeria

## Abstract

Hypoxia-inducible factors (HIFs) are transcription factors that activate the transcription of genes necessary to circumvent to hypoxic (low oxygen level) environments. In carcinogenesis, HIFs play a critical role. Indeed, HIF-1*α* has been validated as a promising target for novel cancer therapeutics, even as clinical investigations have linked increased levels of HIF-1*α* with aggressive cancer progression as well as poor patient prognosis. More so, inhibiting HIF-1 activity restricted cancer progression. Therefore, HIF-1 is a viable target for cancer therapy. This may be expected considering the fact that cancer cells are known to be hypoxic. In order to survive the hypoxic microenvironment, cancer cells activate several biochemical pathways via the HIF-1*α*. Additionally, cellular and molecular insights have proved prospects of the HIF-1*α* pathway for the development of novel anticancer treatment strategies. The biochemical importance of hypoxia-inducible factors (HIFs) cannot be overemphasized as carcinogenesis, cancer progression, and HIFs are intricately linked. Therefore, this review highlights the significance of these linkages and also the prospects of HIFs as an alternative source of cancer therapies.

## 1. Introduction

The function and survival of living organisms are dependent on the adequate supply of oxygen available to the cells. Animals catabolize the sugar from plant using glycolysis, citric acids, and oxidative phosphorylation in an aerobic state. During these processes, oxygen is used as an electron acceptor. Inefficient transfer of electron results in a risk of oxygen species generation. Electron escape can lead to generation of superoxide anion and/or hydroxyl radicals, all of which are examples of reactive oxygen species (ROS) [1]. ROS have the potential to destroy the configuration of biomolecules, which could result in cellular damage or cell death [2]. Increased ROS production is associated with deviations from physiological oxygen pressure (PO_2_) in the electron transport chain. Therefore, tight regulation of cellular oxygen concentration through homeostatic mechanisms is very essential. When the supply of oxygen fails to meet the demand from tissues and cells, it is called hypoxia. All solid tumors are characterized by hypoxia, as proliferation of the tumor cells results in deprivation of oxygen due to insufficient blood flow from abnormal tumor microvasculature. Hypoxia induces stress in organisms either through pathological or through nonpathological conditions [3].

The consequences of deregulation of hypoxia in cells include breakage of DNA strand, oxidative DNA damage, and gene aberration which hinder cell growth and eventual cellular death. It also affects the development of diseases such as chronic lung disease, cancer, diabetes, ischemic heart diseases, stroke, and advanced atherosclerosis [4]. Hypoxia signaling adaptation in a cell is facilitated by the transcriptional regulation family called hypoxia-inducible factor (HIF). HIF is an oxygen-labile DNA-binding transcriptional activator [5]. HIF controls multiple gene expression involved in a process of cancer cell adaptation and progression [6]. Therefore, a better understanding of the molecular mechanism of hypoxia in cancer cells could afford the development of more effective therapy for solid tumors [7]. Additionally, the available cancer therapies have not been desirably effective [8], thus making research efforts aimed at identifying and developing newer cancer treatment strategies imperative. In light of this, the current review is aimed at discussing the prospects of hypoxia-inducible factors as alternative treatment strategy for cancer.

## 2. Cellular Response to Hypoxia

Hypoxia can simply be defined as a state of decreased oxygen level in the cell or tissue when the oxygen provided for use in the tissue is far less than what is actually needed. A cell can be said to be hypoxia when the overall oxygen pressure in the cell is less than 40 mmHg [9]. Oxygen is extremely important, especially in the cells and tissues of mammals, mostly because of its importance in respiration; it is extremely necessary in the final step of the electron transport chain, as the final electron acceptor in oxidative phosphorylation. Its presence increases the chances of reactive oxygen species (ROS) generation, which react with other biological molecules, resulting in the alteration of the biochemical and physical properties of the cell, causing either an upset of the delicate functions or cell death [1]. Therefore, it can be seen that hypoxia is a potentially lethal condition, for both the cell and the tissue at large, if it is allowed to persist long enough. It causes the oxygen-dependent process of respiration to either slow down or cease completely, particularly the oxidative phosphorylation process, which transfers the chemical energy stored in C-H bonds into the high-energy inorganic phosphate bonds found in ATP [10].

The stoppage of oxidative phosphorylation causes a decrease in ATP, ultimately leading to the stoppage of the ATP-dependent sodium potassium pump. This leads to an imbalance of ions, creating an unbalanced cell environment; the stoppage of the sodium potassium pump forces the cell into employing anaerobic respiration as a means of survival, as its oxygen is cut off. This causes a buildup of lactic acid in the cell, and the pH level drops, as the cell becomes increasingly acidic. The acidity of the cell causes it to swell, as it absorbs water from the environment in an attempt to stabilize its pH. When the cell swells, the permeability of the plasma membrane increases, allowing the leaking of soluble enzymes and coenzymes. If hypoxia persists, the continuing depletion of ATP leads to more serious and pronounced structural anomalies. The comprehensive cellular structure is upset, resulting in loss of the extracellular characteristics such as microvilli, and irregular bulges are formed in the plasma membrane of the cell and eventual cellular death.

Mammals have different mechanisms for surviving the events of hypoxia. The human response to a condition of hypoxia involves physiological changes in respiratory, hematopoietic, and cardiovascular systems. The intake of oxygen is maximized by increasing the functions of the cardiac systems, while the rate of oxygen distribution to individual cells is improved by the acceleration of erythropoiesis. At the cellular level, however, there are intricate factors that underlie these physiological changes in response to hypoxia. These factors seek to restore the oxygenation, minimizing hypoxic environment. These intricate factors are known as hypoxia-inducible factors (HIFs) [11, 12].

## 3. Hypoxia-Inducible Factors (HIFs)

Hypoxia-inducible factor 1 is the heterodimer protein of two subunits: HIF-1*α* and HIF-1*β* transcriptional factor [13, 14]. Each contains helix-loop- (HLS-) PER-ARNT-SIM (HLS-PAS) domains that facilitate DNA binding and heterodimerization. The beta subunit can also be referred to as the aryl hydrocarbon receptor nuclear translocator (ARNT). The alpha subunit is sensitive to oxygen, while the *β* subunit (HIF-1*β*) is oxygen dependent [4]. HIF transcription factor is the master regulator of the translational response, and it is produced as a result of oxygen deficiency in the cell [1, 6]. HIF-*α* consists of different alpha subunits: HIF-1*α*, HIF-2*α*, and HIF-3*α*. The *α* subunit of HIF is tightly regulated by HIF prolyl hydroxylases (PHDs). PHDs hydroxylate specific prolyl residues at the HIF-*α* subunits. von Hippel-Lindau (VHL) tumor suppressors E3 ligase recognize hydroxylated HIF-*α* subunit for degradation [4, 15]. In addition, there is reduced transcriptional activities when the factor inhibiting HIF (FIH) hydrolyses HIFs. Decreased activities of PHD and FIH stabilize HIF-*α* during hypoxia, leading to its translocation to the nucleus, where it subsequently binds with HIF-*β* to form a complex. This complex then binds target genes containing the hypoxia responsive element and transactivates the gene expression for different signaling pathways [4]. Essentially, HIF-1 can be referred to as a messenger which migrates towards the nucleus to activate transcription responses to hypoxia. HIF-1 has been involved in gene regulation involving metastasis, growth, tumorigenesis, angiogenesis, and invasion.

The vascular endothelial growth factor (VEGF) is an example of the HIF-1 target gene in which its expression is induced by hypoxia. Meanwhile, HIF-1 alone does not determine the specific gene expression by individual cells, as this is relatively determined by the functional interaction of HIF-1 with other transcriptional factors that control the activation of a selected subgroup of HIF-1 in hypoxic cells [13].

## 4. General Functions of Hypoxia-Inducible Factors (HIFs)

**Figure 1:**
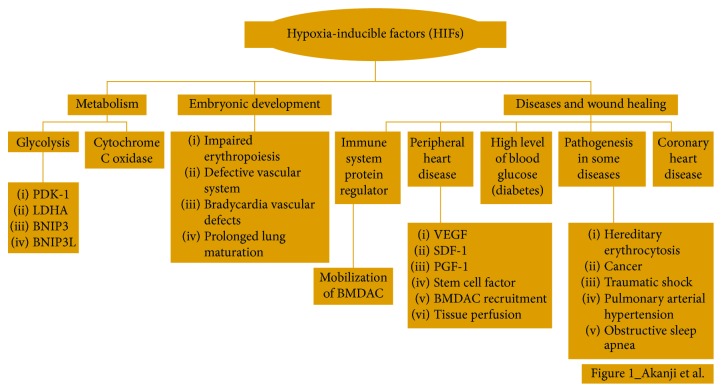
The general function of hypoxia-inducible factors (HIFs).

HIFs perform very essential roles, in a vast number of mammalian conditions and reactions (Figure 1), and any form of impairment of their functions can result in dire consequences. Briefly, few studies that highlight the roles of the HIFs are described in the following.

### 4.1. Metabolism

- HIF-1*α* has been shown to cause a transition from oxidative to glycolytic metabolism by inducing the transcription of genes which support glycolytic metabolism, PDK-1, coding for pyruvate dehydrogenase kinase-1, which inactivates pyruvate dehydrogenase, inhibiting the reaction converting pyruvate dehydrogenase, inhibiting the reaction converting pyruvate to acetyl-CoA, preventing subsequent continuation into the Krebs cycle [16, 17], LDHA, which encodes for lactate dehydrogenase, that catalyzes the reaction converting pyruvate to lactate [18], BNIP3 and BNIP3L, which mediate mitochondrial autophagy [19, 20]
- HIF-1 also mediates a change in the proteinous configuration of the enzyme cytochrome c oxidase, facilitating improvement in the transfer of electrons in situations of hypoxia [21]

### 4.2. Embryonic Development

Most of the discoveries made concerning the role of HIFs in embryonic development were obtained from experiments conducted on mice by quite a number of scientists. From these experiments, it was discovered that

- the circulatory system depends on HIFs for normal development. For example, mouse embryos defective in the gene coding for the HIF (HIF-1*α* precisely) died on their 11^th^ day due to impaired erythropoiesis and defective vascular system [22]
- mouse embryos which died on the 13^th^ day due to bradycardia or vascular defects are usually defective in the genes coding for HIF-2*α* [23, 24]
- neonate mice may also die at birth due to prolonged lung maturation or at a few months after birth, due to reactive oxygen species- (ROS-) mediated organ failure, showing a role of HIFs in organ development [25]
- increased HIF concentration in fetuses due to a reduced blood flow, which brings about a state of prolonged hypoxia, could cause congenital malfunctions

### 4.3. Diseases and Wound Healing

HIFs mediate protective responses activated by the immune system response to disease or injury.

- In coronary heart disease, adenosine is extremely important as it mediates preconditioning, an initial immune response to hypoxia, where exposure of the heart to short periods of hypoxia is followed by reperfusion, protecting the heart against subsequent, long episodes of hypoxia. HIFs activate the transcription of the genes that code for adenosine, which offshoots the aforementioned process [26]
- In the process of healing wounds, HIFs regulate the release of regulatory protein of the immune system from the wound which facilitates the mobilization and direction of bone marrow-derived angiogenic cells (BMDACs) to the site of the wound. BMDACs then stimulate vasculogenesis or angiogenesis, enabling the wound to heal [27]
- The effect of HIFs in wound healing was found to be impaired in mice with a high level of blood glucose. It can be said that diabetes inhibits the action of HIFs [28, 29]
- In peripheral heart disease (PAD) of which limb ischaemia is a complication, HIFs mediate the activation of various target genes which encode for multiple angiogenic growth factors, including the vascular endothelial growth factor (VEGF), stromal-derived factor-1 (SDF-1), placental growth factor (PGF-1), and stem cell factor. HIFs also oversee the recruitment of BMDACs and recover tissue perfusion
- HIFs also contribute to pathogenesis in some diseases like hereditary erythrocytosis [22], cancer [30], traumatic shock, pulmonary arterial hypertension, and obstructive sleep apnea [27]

## 5. Regulation of HIFs

**Figure 2:**
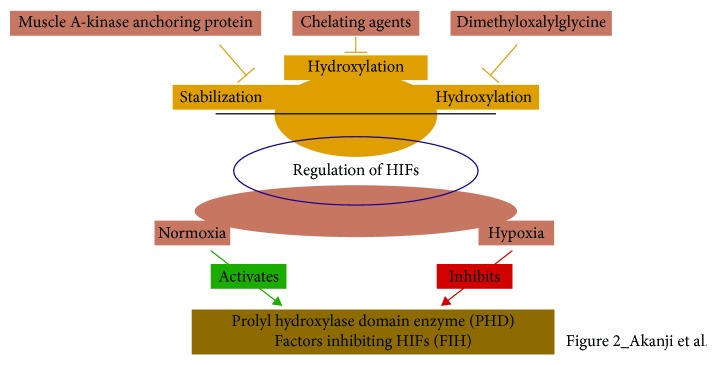
The regulation of HIFs by various cellular factors—cell proliferation and survival, invasion and metastasis, epithelial-mesenchymal transition, metabolic programming, and angiogenesis.

The expression of HIFs in the cellular environment is a closely regulated process, where a lot of factors and reactions are involved (Figure 2). Since HIFs have mainly to do with oxygen levels in tissues, their system of regulation can be considered under conditions of normal oxygen pressure (normoxia) and conditions of abnormal oxygen pressure (hypoxia).

In normoxic conditions, the HIF expression is constitutive or rather inhibited, as they are not required. HIFs are regulated in normoxic conditions by special oxygen sensitive enzymes called prolyl hydroxylase domain enzyme (PHDs). These enzymes regulate HIFs by hydroxylating the proline residues found in the oxygen-dependent degradation (ODD) domain of HIFs [31, 32]. The hydroxylation is carried out by inserting an oxygen molecule into proline and another into *α*-ketoglutarate, splitting it into succinate and carbon dioxide. Since the PHDs use oxygen as a substrate, if oxygen is not available, the process cannot take place [33]. The hydroxylation process is a precursor to another very important step, which is the ubiquitylation of the HIF by von Hippel-Lindau (VHL) protein. The *β* subunit of the VHL protein recognizes and binds the newly hydroxylated HIF. The *α* subunit of the pVHL then assembles the pVHL ubiquitin ligase, which marks the HIF for cleavage by the 26S proteasome [34].

HIFs are also regulated by factors inhibiting HIFs (FIHs) in normoxic conditions. They function by repressing the transactivation of the HIF-*α* subunit. They do this by hydroxylating asparagine residues in the C-terminal transactivation domain of the HIFs using oxygen and *α*-ketoglutarate as reactions, thus preventing the interaction of the hypoxia-inducible factor with the p300 coactivator protein [35, 36].

In hypoxic conditions, however, most of the above processes are reversed. The PHDs, for instance, require oxygen in order to hydroxylate the protein residues. The hydroxylation of the HIF is thus stopped under conditions of hypoxia, making it impossible for it to be recognized and marked for degradation by the pVHL ubiquitin ligase complex. As a result, HIFs are accumulated in the nucleus.

The FIH-mediated hydroxylation is also reduced in the conditions of hypoxia, allowing the HIFs react with the transcriptional coactivators p300/CREB-binding protein [37]. This transcriptional complex that is activated leads to the transcription of a particular set of genes, as a part of the cellular response to hypoxia, which includes, but is not limited to, SLC2A1 (glycolysis) and VEGFA (angiogenesis) [38].

HIFs may also be regulated in some other ways as follows:

- Muscle A-kinase anchoring protein (mAKAP): AKAPs are scaffolding proteins that mediate the assembly of multiprotein complexes. The mAKAPs arrange the E3 ubiquitin ligase complex, affecting the stability and positioning of HIF in the active site of the enzyme. A decrease in the availability of the mAKAP would alter the stability of the HIF complex
- Dimethyloxalylglycine (DMOG) is a well-known opponent of *α*-ketoglutarate, which, if inhibited, would abrogate the function of the hydroxylase, thereby supporting HIF transcription [39]
- HIF is also stimulated by chelating agents of iron, desferrioxamine and cobalt chloride (Adeyemi et al., 2017). These chelators inhibit the hydroxylases by displacing the iron ions present in their catalytic centers
- Doxorubicin (adriamycin) is a chemotherapeutic drug used for cancer treatment. HIF-1 transcriptional activity was inhibited by doxorubicin by preventing the binding of HIF-1 to DNA [40, 41]

## 6. HIF Regulation and Mitochondria Function in Cancer

The tricarboxylic acid (TCA) cycle catalyzes enzymatic reactions that provide electrons in the form of the reducing equivalents NADH and FADH_2_ to the electron transport chain (ETC) in the mitochondrial matrix. Different intermediates enter the cycle at a different point from other pathways but under hypoxia; glucose and fatty acid-derived carbons are diverted from being broken down to acetyl-CoA, while glutamine-derived carbons are diverted from being catabolized to succinyl-CoA by the HIF-regulated genes.

Decreased oxidative phosphorylation could induce HIF to upregulate lactate dehydrogenase (LDHA), thus regenerating NAD to maintain ATP production from glycolysis, and thereby, divert pyruvate from breakdown into acetyl-CoA which adversely suppress both TCA and ETC activities [17].

Cells adapt their metabolic programme under hypoxia to maintain the reactions that rely on ATP produced by oxidative phosphorylation. Generally, HIF-1 signaling supports the production of ATP anaerobic and downregulation of oxidative phosphorylation, thereby reducing the cell's reliance on oxygen-dependent energy production [17]. In relation to mitochondrial function, it has been noted that the coexpression of HIF-1*α* and HIF-2*α* has some opposing roles; however, they both in a similar manner decrease a cell's dependence on mitochondrial oxidative phosphorylation [42].

Stress signaling pathways in the cell-like hypoxic response [43], redox signaling [44], and unfolded protein response [45] are activated in the mitochondria. As evident in previous studies using mitochondrial DNA- (mtDNA-) deficient *ρ*0 cells in mouse xenograft models, it was observed that the growth of the tumor is accelerated by the mitochondria (Tan, et al., 2015; Yan et al., 2015). Cancer prognosis has been linked clinically to single nucleotide variants in mtDNA [46, 47]. However, mtDNA mutations or reduced mitochondrial content has caused decreased or low mitochondrial function noticeable in many cancer types, including pancreatic, kidney, thyroid, and colon cancer [48–50]. This suggests that there are some adaptive mechanisms during tumor development in which mitochondrial activity is decreased.

## 7. MicroRNAs and Cancer

MicroRNAs also called miRNAs or miRs are small noncoding RNAs which regulate gene expression at the posttranscriptional level. miRNAs repress mRNA translation and degrades RNA targets [51]. miRNAs give a new insight into cancer studies. miRNA genes are an important factor in the pathogenesis of human cancer as they form central nodal points in cancer development [52]. Understanding the mechanistic role of miRNAs in cancers still presents a challenge. Reports have shown that molecular pathways of cancer are regulated by miRNAs by targeting oncogenes and tumor suppressor genes, involving the cancer-stem-cell development pathway, angiogenesis, and drug resistance [53].

## 8. HIF-1 Responses in Tumor and Prospects for Targeted Therapies

Tumors are noticeably characterized by a low oxygen level of the tumor microenvironment. A partial pressure (PO_2_) of less than 10 mm is exhibited in solid tumors compared to 45-65 mm in normal tissues. There is inadequate blood perfusion in acute or transient hypoxia, but chronic hypoxia limits diffusion of oxygen in enlarged tumors. This leads to the activation of both HIF-1 and HIF-2 with overexpression of HIF-1*α* which is linked to metastasis and mortality [4].

Cancer cells in humans have overexpression of HIF-1*α*, but this is dependent on the type of cancer. The overexpressed HIF-1*α* has resulted into high mortality rate in patient experiencing cancers of the breast, ovary, uterus, cervix, brain, and oropharynx, while overexpression of HIF-1*α* has been associated with decreased death rate with head and neck cancer patients [54]. Although studies have indicated that HIF-1*α* facilitates resistance to radiation and chemotherapy, the inhibition of HIF-1*α* activation may be useful in hindering cancer progression, thereby starving the growing tumor cell of oxygen and the required nutrient supply [54].

## 9. HIFs in Cancer Progression

**Figure 3:**
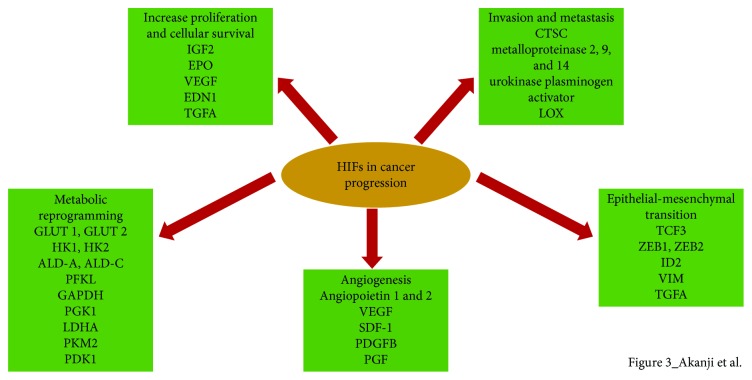
HIFs in cancer progression.

The significances of HIFs in different stages in cancer cell formation cannot be overemphasized (Figure 3). The different stages include angiogenesis, metastasis, metabolic reprogramme, invasion, epithelial-mesenchymal transition, and cell proliferation and survival. With different clinical and experimental research establishing HIF as a cancer therapy target, HIF-1*α* and HIF-2*α* levels are associated with metastasis, vascularization, and tumor growth in both animal-based and clinical-based studies. Several HIF-regulated genes that are identified as important in cancer development are as follows [1].

- Increase proliferation and survival of cell: a major distinction between tumor cell and normal cell which is initiated by autocrine signaling increased cell proliferation and reduced cell death. The level of ATP is an important determinant of cell apoptosis as abundant glycolytic ATP leads to apoptosis during hypoxic. Besides, deprivation of oxygen leads to the inhibition of or decreased electron transport chain processes, thus reducing the mitochondrial membrane potential [55]. This results in the activation of survival/growth factors which are expressed by HIF-regulated genes such as insulin-like growth factor-2 (IGF2), erythropoietin (EPO), vascular endothelial growth factor (VEGF), Endothelin 1 (EDN1), transforming growth factor-*α* (TGFA), and adrenomedullin [1]. These genes are the controlling hub of tumor pathways such as invasion, proliferation, angiogenesis, and colonization of far-off sites [5]
- Metabolic reprogramming: in order to meet cell energy demands, glucose uptake is highly upregulated in cancer cells compared to a normal cell. It is the basis to detect metastases by imaging using 18-F-fluorodeoxyglucose-positron emission tomography (FDG-PET). HIF-1 also moderate the tumor-related metabolic switch through the Warburg effect which is responsible for greater glucose oxidation in anaerobic condition than in oxidative phosphorylation. Critical effects of this shift are tumor microenvironment acidosis. The acidic environment and the metabolic switch are responsible for abundant metabolic intermediates that stimulates tumor progression and aggressiveness [55]. HIF-1 facilitates the gene expression encoding glucose transporter 1 and 3 and enzymes involved in glucose conversion to lactose different from those found in normal cells such as hexokinase 1 and 2 (HK1, HK2), aldolase A and C (ALD-A, ALD-C), phosphofructokinase L (PFKL), glyceraldehyde-3-phosphate dehydrogenase (GAPDH), enolase A, phosphoglycerate kinase 1 (PGK1), lactate dehydrogenase A (LDHA), and pyruvate kinase M2 (PKM2). HIF-1 increases the pyruvate dehydrogenase kinase-1 (PDK1) expression which inhibits pyruvate dehydrogenase responsible for converting pyruvate to acetyl-CoA before TCA cycle can occur, thereby suppressing mitochondria function and oxygen utilization [1]. Hexokinase and lactate dehydrogenase A are oncogenic transcription factors for MYC targets. Under physiological condition, c-MYC activities are inhibited by HIF-1*α*, but both c-MYC and HIF-1*α* work hand in hand to induce the pyruvate dehydrogenase kinase-1 (PDK1) and hexokinase expression which result in aerobic glycolysis and angiogenesis. Additionally, HIF-1*α* influences cytochrome c oxidase subunit 4 (COX4) switch under hypoxic condition to give a homeostatic response which improves respiration efficacy at different oxygen concentrations [5]. Semenza [27] also revealed that HIF-1 may also mediate the transketolase enzyme expression in the hexose monophosphate pathway required for nonoxidative production of ribose, a precursor for nucleic acid [5, 27]
- Angiogenesis: new capillaries formed from already existing vessels in response to low oxygen especially in cancerous cell to deliver oxygen to the cells and thereby encourage tumor growth [56]. The angiogenic switch regulated by HIF-1 in hypoxic tumor microenvironment may be connected with increased oxygen consumption, while reducing oxygen diffusion distance. Angiogenesis is an intricate, well-ordered process which is essential for neoplasm formation. The mechanism comprises of many genes, regulators, and pathways. Induction of angiogenesis results in enlarged vascular density and reduced oxygen diffusion distance [5]. Furthermore, HIF-1 also regulates the encoding genes for angiogenic growth factor expressions. These include angiopoietin 1 and 2, vascular endothelial growth factor (VEGF), stromal-derived factor-1 (SDF-1), platelet-derived growth factor B (PDGFB), and placenta growth factor (PGF) [27]. A critical link between hypoxia and angiogenesis is the discovery of vascular endothelial growth factor (VEGF)
- Epithelial-mesenchymal transition: HIF-1 triggers activation of repressor genes that inhibit proteins responsible for cell to cell contact and rigid cytoskeleton. Examples of such repressor genes include transcription factor 3 (TCF3), zinc finger E-box-binding homeobox 1 and 2 (ZEB1, ZEB2), and inhibitor of differentiation 2 (ID2). HIF-1 also facilitates a gene expression that stimulates flexible cytoskeleton like TGFA and vimentin (VIM) [1]
- Invasion and metastasis: invasion and metastasis of tumor cells are regulated by hypoxia. Metastasis is a series of well-defined events which is the basic reason of cancer-related mortality. These events include the local spread of tumor cells, intravasation, survival of circulating tumor cells, and extravasation followed by proliferation that leads to colonization. Activation of genes regulated by HIF may improve metastasis in multiple tumors. HIF-1 stimulates genetic transcription such as proteases that degrade cathepsin C (CTSC), matrix metalloproteinase 2, 9, and 14, and the urokinase plasminogen activator receptor or remodel lysyl oxidase (LOX); the extracellular matrices within are of metastasis [5]

## 10. Inhibitors of HIF-1 in Cancer Therapy

Different chemical compounds or drugs have been revealed to block the activity of HIF through different molecular mechanisms (Table 1), including a reduced synthesis of HIF-1*α* protein (mTOR inhibitors, cardiac glycosides, topoisomerase inhibitor, and synthetic oligonucleotides), decreased HIF-1*α* mRNA levels (aminoflavone component of prodrug AFP-464), increased HIF-1*α* breakdown (HSP90 inhibitors, antioxidants, and Se-methylselenocysteine), reduced heterodimerization of HIF subunit (acriflavine), decreased DNA binding to the HIF (anthracyclines and echinomycin), and reduced transcriptional activity [1].

- Inhibitors of the HIF-1 mRNA expression: HIF-1 increase is regulated predominantly at the degradation or translation of protein, and these pathways are the targets of most HIF-1 inhibitors. However, under hypoxic conditions, HIF-1 mRNA levels can act as a limiting factor thereby affecting protein translation [57]. Aminoflavone (AF) is an agent that affects the HIF-1 mRNA expression. It acts as a ligand of aryl-hydrocarbon receptor (AhR) and presently being used in clinical trials in metastatic cancer patients [57]
- Inhibitors of HIF-1 protein translation: numerous agents may affect the HIF-1 protein synthesis rate, including tyrosine kinase inhibitor, topoisomerase I and II inhibitor, cyclin-dependent kinase inhibitor, oncogenic pathway inhibitor, and thioredoxin reductase inhibitor. One of the earlier agents used for HIF-1 protein translation is topotecan, a second line chemotherapy for lung cancer or ovarian cancer. Topotecan is a camptothecin analogue which in the presence of DNA replication generate double strand DNA breaks and cytotoxicity, thereby poisons topoisomerase I by inducing the formation of stable Top1-DNA cleavage complexes [57]. Another class of agents that affect HIF-1 protein translation is cardiac glycosides. Digoxin in particular has been identified as a HIF-1 potent inhibitor. Digoxin inhibits HIF-1 translation using mTOR-independent mechanism and also exhibits antitumor activity [55]. PX-478 is another HIF-1 inhibitor presently in phase I clinical trials in advanced metastatic cancer patients. It showed antitumor activity in tumor xenograft models, which correlate with the HIF-1 expression [57]

EZN-2968 is a RNA modulator composed of synthetic antisense oligonucleotide that binds and inhibits specifically the HIF-1*α* mRNA expression [6, 58]. There is a dose-dependent downregulation of HIF-1*α* mRNA after it binds to EZN-2968 leading to inhibition in both normoxia and hypoxia [58]. In mice implanted with DU-145 human prostate cancer cells, EZN-2968 treatment showed tumor reduction. Clinically, evaluation of EZN-2968 treatment of 4 out of 6 patients with paired tumor biopsies showed reduced HIF-1*α* mRNA in posttreatment biopsies while two patients had a reduced level of mRNA and HIF-1*α* protein of target genes in biopsies [59]. This revealed a pilot proof of HIF-1*α* mRNA and protein expression modulation in response to EZN-2968 thereby indicating inhibition of HIF-1*α* mRNA has potential as a target for cancer therapy [60].

## 11. Conclusion

The mechanism for cellular oxygen homeostasis and its response to a low oxygen state is basically facilitated by the HIF pathway. Additionally, the regulation or dysregulation of the HIF pathway is a major determinant in cancer metastasis, and this correlates with a poor cancer prognosis. Because of the roles that it plays in cancer progression, HIF has become an attractive target for chemotherapy against cancerous cells. Perhaps, the combined usage of conventional treatment and HIF inhibitors may prove to be useful clinically.

## Figures and Tables

**Table 1:** Some HIF inhibitors and targets.

Agents	Inhibitory mechanisms	Targeting HIF-1	Targeting HIF-2	Reference no.
EZN-2968	HIF-1 mRNA expression	√	—	Jeong, et al., [60]
EZN-2208	HIF-1 mRNA expression	√	—	Coltella, et al., [61]
Topotecan	HIF-1 mRNA translation	√	—	Rapisarda, et al., [62]
HIF-1*α* inhibitor	HIF-*α* transcriptional activity	√	√	Cui, et al., [63]
PX-12	HIF-*α* transcriptional activity	√	—	Raninga, et al., [64]
Acriflavine	HIF transcriptional activity	√	√	Lee, et al., [40, 41]
Echinomycin	HIF DNA binding	√	—	Yu, et al., [65]

## Abbreviations

ALD-A and ALD-C: Aldolase A and aldolase C
AF: Aminoflavone
ARNT: Aryl hydrocarbon receptor nuclear translocator
AhR: Aryl-hydrocarbon receptor
BNIP3: BCL2/adenovirus E1B 19-kDa interacting protein 3
BNIP3L: BNIP3-like
BMDACs: Bone marrow-derived angiogenic cells
CTSC: Cathepsin C
COX4: Cytochrome c oxidase subunit 4
DMOG: Dimethyloxalylglycine
ETC: Electron transport chain
EDN1: Endothelin 1
EPO: Erythropoietin
FDG-PET: 18-F-fluorodeoxyglucose-positron emission tomography
FIH: Factor inhibiting HIF
GAPDH: Glyceraldehyde-3-phosphate dehydrogenase
HK1 and HK2: Hexokinase 1 and 2
ID2: Inhibitor of differentiation 2
IGF2: Insulin-like growth factor-2
LDHA: Lactate dehydrogenase A
LOX: Lysyl oxidase
miRNAs: MicroRNAs
mAKAP: Muscle A-kinase anchoring protein
ODD: Oxygen-dependent degradation
PO_2_: Partial oxygen pressure
PAD: Peripheral heart disease
PFKL: Phosphofructokinase L
PGK1: Phosphoglycerate kinase 1
PGF: Placenta growth factor
PGF-1: Placental growth factor
PDGFB: Platelet-derived growth factor B
PHD: Prolyl hydroxylases
PDK1: Pyruvate dehydrogenase kinase-1
PKM2: Pyruvate kinase M2
ROS: Reactive oxygen species
SDF-1: Stroma-derived factor-1
TCF3: Transcription factor 3 (TCF3)
TGFA: Transforming growth factor-*α*
TCA: Tricarboxylic acid
VEGF: Vascular endothelial growth factor
VIM: Vimentin
VHL: Von Hippel-Lindau
ZEB1 and ZEB2: Zinc finger E-box-binding homeobox 1 and 2.
